# Emerging and Innovative Theranostic Approaches for Mesoporous Silica Nanoparticles in Hepatocellular Carcinoma: Current Status and Advances

**DOI:** 10.3389/fbioe.2020.00184

**Published:** 2020-03-10

**Authors:** Yaoye Tao, Jianguo Wang, Xiao Xu

**Affiliations:** ^1^Division of Hepatobiliary and Pancreatic Surgery, Department of Surgery First Affiliated Hospital, School of Medicine, Zhejiang University, Hangzhou, China; ^2^National Health Commission (NHC) Key Laboratory of Combined Multi-Organ Transplantation, Hangzhou, China; ^3^Key Laboratory of the Diagnosis and Treatment of Organ Transplantation, Chinese Academy of Medical Sciences (CAMS), Hangzhou, China; ^4^Key Laboratory of Organ Transplantation, Hangzhou, China

**Keywords:** mesoporous silica nanoparticles, hepatocellular carcinoma, theranostic, precision delivery, biomedical applications

## Abstract

Hepatocellular carcinoma (HCC) is one of the most prevalent and lethal solid cancers globally. To improve diagnosis sensitivities and treatment efficacies, the development of new theranostic nanoplatforms for efficient HCC management is urgently needed. In the past decade, mesoporous silica nanoparticles (MSNs) with tailored structure, large surface area, high agents loading volume, abundant chemistry functionality, acceptable biocompatibility have received more and more attention in HCC theranostic. This review outlines the recent advances in MSNs-based systems for HCC therapy and diagnosis. The multifunctional hybrid nanostructures that have both of therapy and diagnosis abilities are highlighted. And the precision delivery strategies of MSNs in HCC are also discussed. Final, we conclude with our personal perspectives on the future development and challenges of MSNs.

## Introduction

Liver cancer is currently the fourth primary cause from cancer-related deaths and its incidence and mortality is still increasing, with an estimated 841,080 new cases and 781,631 deaths from this disease in 2018 (Llovet et al., 2016; Bray et al., 2018; World Health Organization [WHO], 2018). Among all primary liver cancers, hepatocellular carcinoma (HCC) represents approximately 90% of all cases. Surgical resection and liver transplantation are considered the curative therapies for long-term control of HCC, however, the majority of HCC patients are diagnosed at advanced stages beyond the standard of surgical treatment (GBD, 2013; Roberto et al., 2016; Yegin et al., 2016). A few molecular targeting drugs such as sorafenib (SO) approved for advanced HCC, which show merely a marginal survival benefit contrasting with conventional drugs. Unfortunately, its efficacy and adverse effects for HCC patients remained unsatisfactory (Bruix et al., 2015; Gao J. et al., 2015). Therefore, new treatment and diagnose modalities for the management of HCC are urgently warranted.

**FIGURE 1:**
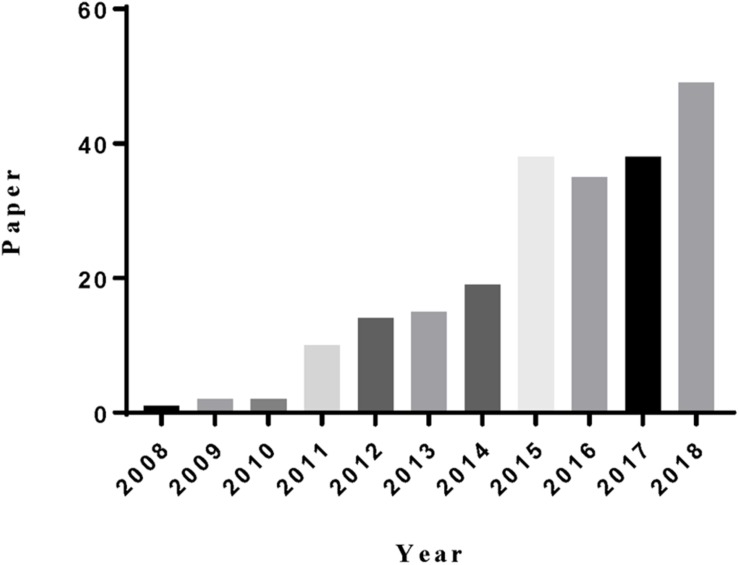
The statistics of the paper indexed in the ISI web of science by the topic of “mesoporous silica” and “liver cancer.”

**FIGURE 2:**
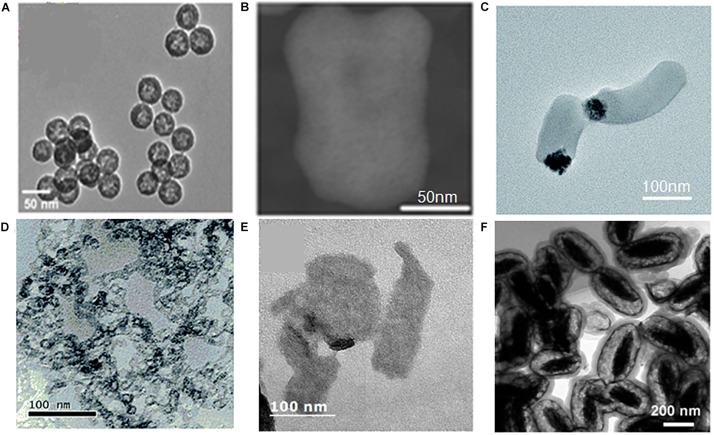
TEM of samples **(A)** (MSNs-1), **(B)** (MSNs-2), **(C)** (MSNs-3), **(D)** (MSNs-4), **(E)** (MSNs-5), **(F)** (MSNs-6) with different size and shape. **(A)** Reproduced with permission from Li et al. (2018b). **(B)** Reproduced with permission from Li et al. (2018c). (C) Reproduced with permission from Xing et al. (2018). **(D)** Reproduced with permission from Liu et al. (2017). **(E)** Reproduced with permission from Lee et al. (2016). **(F)** Reproduced with permission from Lv et al. (2015).

With the development of nanotechnology, nanomaterials modified as multifunctional nanoplatforms for cancer therapeutics, diagnostics, or both (known as “theranostics”) attracted increasing attention (Wang J. et al., 2016; Xu et al., 2018; Kesse et al., 2019). Since the first report using silica nanoparticles (Cornell Dot) accepted by the United States Food and Drug Administration (FDA) for a stage-I human clinical trial in 2011 (Benezra et al., 2011), the recent decade has witnessed a steadily increase in research on biomedical application of mesoporous silica nanoparticles (MSNs) in the liver cancer (Figure 1). In general, MSNs show the following unique structural and biomedical properties:

(a) Adjustable pore size. The tunable pore diameters of MSNs from 2 to 30 nm allow a variety of agents encapsulated in nanoparticles (Kobler and Bein, 2008; Keasberry et al., 2017). Moreover, hierarchically MSNs which simultaneously consist of large pores and small pores throughout the whole particle are more effective for the diffusion of two different guest molecules in one unit (Jin et al., 2014).
(b) Tunable particle size and shape. The particle size of MSNs can be controlled from 10 to 1000 nm, and the particle morphology can be controlled from rod-, sphere-, to wormlike structures (Figure 2; Huh et al., 2003; He et al., 2009). MSNs with different size and shape have unique characteristic (Lu et al., 2009; Meng et al., 2011b), which is convenient for researchers choosing the most suitable particle to achieve their aims.
(c) Ordered mesoporosity and large surface area. The ordered mesoporous structure with disjoint between individual porous channels enable better control of agents loading and release kinetics (Hu et al., 2011). And due to extensive porous structure, MSNs usually have a large surface area enhancing nanoparticles dissolution.
(d) High agents loading volume. Highly porous interior structure ensure a high agent payload of MSNs, usually above 200 mg, maximally about 600 mg agent per 1 g silica (He et al., 2010a). In addition, MSNs synthesized with a hollow core called hollow-type MSNs are capable of encapsulating a super-high dose of agent, typically more than 1000 mg agent per 1 g silica, which is obviously higher than those by other nanoparticles (Zhu et al., 2005a, b).
(e) Facile functional surfaces. MSNs, generally speaking, have two functional surfaces, namely exterior particle surface and cylindrical pore channel surface. However, for hollow-type MSNs, there is an extra interior particle surface. These surfaces can be easily functionalized by virtue of the silane coupling chemistry (He et al., 2010b; Cheng W. et al., 2017). Furthermore, both of exterior and interior particle surfaces which could appropriately connect and coat with other materials become the key of creating high-performing hybrid materials (Castillo and Vallet-Regi, 2019).
(f) Excellent biocompatibility. Silica is considered as “Generally Recognized As Safe” (GRAS) by the FDA (ID Code: 14808-6). He et al. discover that MSNs exhibit a three-stage degradation behavior in simulated body fluid, and almost completely degrade in 15 days (He et al., 2010a). Recently several *in vivo* biosafety evaluations of MSNs have been reported (Liu T. et al., 2011; Fu et al., 2013; Choi et al., 2015), indicating MSNs have low *in vivo* toxicity and can be excreted from the body through feces and urine.

These distinctive features endow MSNs with unique advantages to encapsulate a variety of therapeutic and bioimaging agents and implement the desired functions. To give an overview of recent progress of MSNs in theranostic for HCC, this review is arranged as follow. Firstly, it outlines precision delivery strategies of the agents in MSNs to HCC sites and cells. Next, the current state of the research of using MSNs in the field of HCC theranostics are highlighted. Finally, we vision the future advancements for MSNs.

## Precision Delivery Strategies of MSNs in HCC

**FIGURE 3:**
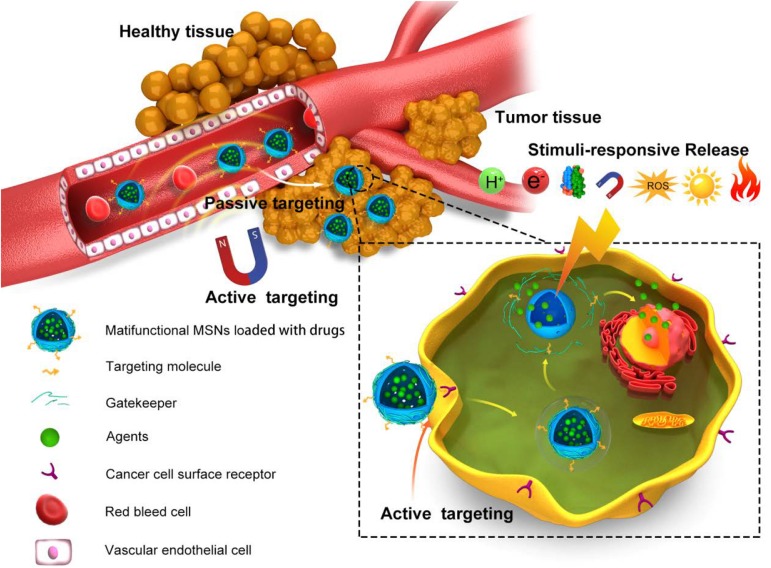
Schema of the delivery strategies of MSNs in HCC. *In vivo* process of precision delivery, when MSNs arrive the vasculature of tumor, passive targeting would first work based on EPR effects. Next, active targeting by conjugation of targeting ligand/receptor and EMF effects would promote MSNs into tumor tissues and cells. Final, stimuli-responsive release in tumor tissues and cells would realize by virtue of pH, redox, light, and so on.

Nanoparticles, designed to deliver agents preferentially to the HCC tissues and cells, provide the precondition for overcoming the shortcomings of conventional treatment and diagnose approaches (Bae et al., 2011). The targeting ability to tumor not only enhances the effects of agents, but also controls dose-limiting side effects in other tissue. A well-designed nanosystem always includes multiple delivery strategies to reach a high accumulation in tumor. In this part, we discuss the delivery strategies developed in MSN-based HCC theranostics (Figure 3).

### Passive Targeting

It is well recognized that liver tumors display remarkable extensive angiogenesis with defective vascular structure, accompanying with impaired lymphatic drainage system (Zhu et al., 2011). Therefore, the vascular networks of HCC have an increased permeability to circulating nanoparticles, while the lymphatic system has a reduced disposal rate to internalized nanoparticles, which allow MSNs to accumulate in HCC tumor interstitial space (Maeda et al., 2000). This so-called enhanced permeation and retention (EPR) effect has been considered as basics for achieving passive targeting in the nanosystems (Maeda et al., 2013).

Particle size, shape, and surface chemistry of MSNs could greatly influence the EPR effect of the nanoparticles (Lee et al., 2009; Maeda et al., 2013; Li et al., 2016). For instance, Meng et al. demonstrated 50 nm MSNs coated with PEI-PEG copolymer yield a intratumoral accumulation of about 12% of the total dose, which is significantly higher compared to 1% of 100 nm phosphonate-coated MSNs and 3% of 50 nm PEGylated MSNs. The additional cationic polymer coated on the MSNs ameliorated the potential downside of PEG surface. In conclusion, size tuning and decoration of the MSNs with PEI-PEG copolymer lead to an dramatic enhancement of EPR effect and sufficient accumulation in tumor (Meng et al., 2011a). Besides, a research from Harvard Medical School showed that combined radiation and cyclophosphamide could enhance tumor-associated vascular leaking, leading to a sixfold increase of nanoparticles accumulation in tumor (Miller et al., 2017).

### Active Targeting

EPR-mediated passive targeting always lacks specificity for different tumor tissues and tumor development stages (Natfji et al., 2017). To improve the targeting efficiency, active targeting strategies have gained much attention recently.

Owing to the overgrowth and abnormality of HCC, many receptors are usually upregulated on the surface of HCC cells, compared to other normal cells. Through the recognition of these receptors by targeting ligands on MSNs, more smart targeting strategies have been achieved. The targeting ligands now used for MSN-based HCC theranostics include lactobionic acid (Zhang et al., 2012), folic acid (Chen et al., 2019), arginine-glycine-aspartate (RGD) (Chen et al., 2012), transferring (Hao et al., 2017), hyaluronic acid (Lee et al., 2018), low-density lipoprotein (LDL) (Ao et al., 2018), and others (Table 1).

**TABLE 1:** Summary of targeting ligands and receptors on MSN-based HCC theranostics.

Ligand	Receptor	Cell type	Animal model	References
Lactobionic acid	Asialoglycoprotein receptor (ASGPR)	HepG2,Huh7, SMMC-7721	HepG2 mice model, H22 mice model	Zhang et al., 2012; Dai et al., 2014; Wang et al., 2017d; Zhao et al., 2017; Pei et al., 2018; Zheng et al., 2018
Folic acid	Folate receptor	HepG2,SMMC-7721	SMMC-7721 mice model, HepG2 mice model, H22 mice model	Chen et al., 2010, 2019; Gao B. et al., 2015; Lv et al., 2015; Wang Y. et al., 2016; Wang et al., 2017a, b; Xu et al., 2017
RGD	Integrin	SMMC-7721, HepG2, Huh7	SMMC-7721 mice model, H22 mice model	Chen et al., 2012; Liao et al., 2014; Yu et al., 2015; Zeng et al., 2016; Fei et al., 2017; Li et al., 2018b
Transferrin	Transferrin receptor	Huh7	N/A	Chen X. et al., 2017; Hao et al., 2017
Hyaluronicacid	CD44	HepG2	N/A	Lee et al., 2018
LDL	LDL receptor	HepG2	HepG2 mice model	Ao et al., 2018
Galactose/lactose	ASGPR	HepG2, SMMC-7721	N/A	An et al., 2015; Quan et al., 2015
SP94	Unknown receptor(s)	Hep3B	N/A	Ashley et al., 2011; Epler et al., 2012
AS1411 aptamer	Nucleolin	HepG2	HepG2 mice model	Zhang et al., 2014
Epithelial cell adhesion molecule (EpCAM)aptamer	EpCAM	HepG2	HepG2 mice model	Babaei et al., 2017
TLS11a aptamer	Unknown receptor(s)	HepG2	N/A	Hu et al., 2017
Glycyrrhetinic acid (GA)	GA receptor	HepG2	N/A	Lv et al., 2017
Cetuximab	Epidermal growth factor receptor (EGFR)	HepG2	HepG2 mice model	Wang J.K. et al., 2017
Tumor necrosis factor-related apoptosis-inducing ligand (TRAIL)	Death receptors 4/5	HepG2	HepG2 mice model	Liu et al., 2017
Anti-CD155/anti-CD112 monoclonalantibodies	CD155/CD112	SMMC-7721, HHCC	SMMC-7721 mice model	Tao et al., 2016
Avidin	Carcinoembryonic antigen (CEA)	Huh7, LM-9	N/A	Chen et al., 2014
Phenylboronic acid	Sialic acid	HepG2	H22 mice model	Tang et al., 2017
Concanavalin A	Glycoprotein receptors	Huh7, ML-1	N/A	Chen et al., 2018
HepG2 cell membranes	Unknown receptor(s)	HepG2	HepG2 mice model	Yue et al., 2018b

In another way, magnetic mesoporous silica nanoparticles (M-MSNs) with superior magnetic properties maintaining the excellent advantages of MSNs could achieve magnetic-mediated targeting functions under external magnetic fields (EMFs) (Shao D. et al., 2016; Wang Y. et al., 2016; Tang et al., 2017). The targeting capacity guided by EMFs including two aspects: on the one hand, M-MSNs would mainly accumulation around the tumor site under the EMFs, which could apply a magnetic force on nanoparticles to enhance the EPR effect and overcome the drag experienced in the blood flow (Thorat et al., 2019a); on another hand, EMFs effect could enhance endocytosis of the tumor cells. Interestingly, under the EMFs about 15% higher fluorescence intensity of nanoparticles was detected in HepG2 cells than that of the non-magnetic field untreated HepG2 cells, while this phenomenon is absence in HL-7702 cells, which means the effect of EMFs is selective for HCC cells (Xing et al., 2018).

### Stimuli-Responsive Release

Nano delivery systems are ideal with “zero premature release” before arriving the disease foci. The stimuli-responsive release can realize this point in response to internal stimuli in tumor microenvironment or external stimuli (Thorat et al., 2019b).

#### Internal Stimuli

**FIGURE 4:**
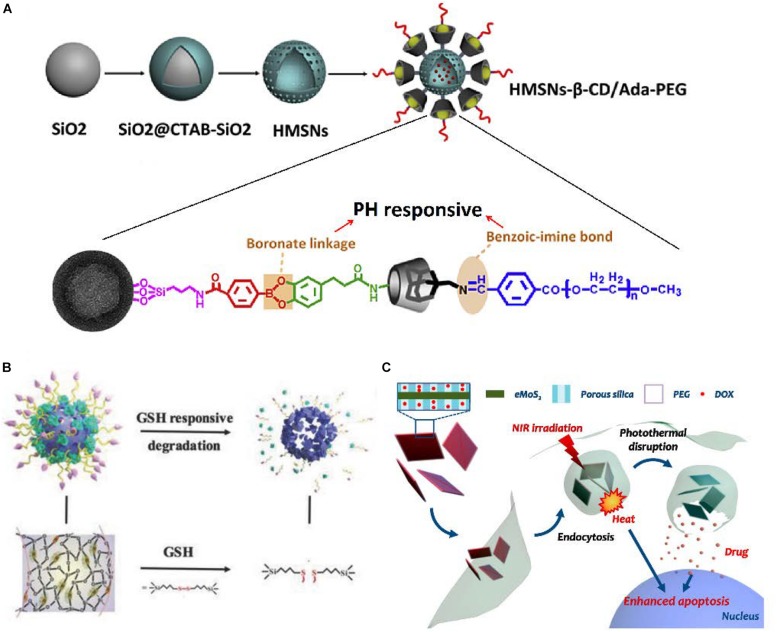
Different stimuli-responsive MSNs. **(A)** Fabrication illustration of dual-pH-responsive MSNs. **(B)** Schematic illustration of MONs. **(C)** Schematic illustration of NIR-responsive MSNs. **(A)** Reproduced with permission from Liu et al. (2016). **(B)** Reproduced with permission from Li et al. (2018b). **(C)** Reproduced with permission from Lee et al. (2016).

Among internal stimuli, PH-responsive release plays the most promising role in HCC, since a more acidic extracellular (pH ≈ 6.8) environment is usually formed around solid tumor tissues than normal tissues and blood (pH ≈ 7.4) due to increased acid production resulting from high glycolysis (Lu et al., 2018). It has been found that electrostatic interactions between the positively charged agents [such as doxorubicin (DOX)] and negatively charged MSNs were greatly reduced by protonation in a low pH condition, which results in a pH-responsive release of DOX (Chen et al., 2018). Similarly, some gatekeepers over the pore entrance to reduce premature release of agents could be protonated under acidic pH, rapidly collapsing, thus the agents would be released from the nanoparticles (Chen et al., 2019). Besides, some chemical bonds connecting the gatekeepers and MSNs, which would be broken down by pH stimulation, are also be used in pH-responsive release (Liu et al., 2016). Differentiated from the common single pH-responsive systems, a novel cascade pH stimuli triggering nanosystem had been developed (Figure 4A). At first, benzoic-imine bonds would be dissociated in the tumor microenvironment pH signal (6.8) to release PEG and improve cellular uptake. Then, boronic acid-catechol ester bonds would be hydrolyzed in the endosome/lysosome pH signal (4.5–6.5) to release more drug in tumor cells, which leads to significant tumor growth inhibition. In tumor-bearing mice, the increased life span of mice treated with this nanoparticles was raised 42.4% than no pH-responsive nanoparticles (Liu et al., 2016).

In addition, HCC cells always undergo oxidative stress, which means the overproduction of various reactive oxygen species (ROS) in tumor cells. In the meantime, HCC cells also have an elevated glutathione (GSH) level to protect themselves under ROS cytotoxicity (Cheng S.B. et al., 2017; Jiang et al., 2017). Consequently, GSH-responsive become one of most popular redox-responsive precision delivery strategies because of the higher GSH level in intracellular matrix of HCC cells than that in extracellular matrix or intracellular matrix of normal cells (Chen X. et al., 2017). The disulfide bond which could be cleaved by GSH is widely used to connect the gatekeepers and MSNs (Chen X. et al., 2017; Tang et al., 2017; Wang J.K. et al., 2017). In one study, the surfaces of MSNs were functionalized by cytochrome c (CytC) via disulfide bonds, which would be rapidly cleaved in HCC cells to release the loaded drug. Around 78.9% of agents was released from MSNs with stimulus of reductive signals after incubation for 3 h, whereas only 5.11% of agents was released for the group without reductive signals (Zhang et al., 2014). Moreover, Yue et al. presented mesoporous organosilica nanoparticles (MONs) containing disulfide bridges inside, synthesized on the framework of MSNs (Figure 4B). In cancer cells with the high concentration of GSH, MONs would be broken into small pieces leading to greater targeting drug release. The fluorescence intensity of DOX in the MONs treated cells was approximately 20% higher than MSNs treated cells (Yue et al., 2018a). In a similar way, the ROS-responsive MSNs are also developed for HCC theranostics (Pei et al., 2018).

In other reports, it has been well established that there is over expression of some enzymes in HCC, such as protease (Menard et al., 2019), glycosidase (Hakeem et al., 2016), and esterase (Xia et al., 2017). Once the enzyme is found at higher level in the tumor site, the MSNs can be programmed to targeting release of agents via enzymatic conversion of the carrier. Some microRNAs such as miR-122 are abundant in liver cancer. Therefore, one type of microRNAs-responsive MSNs in Huh7 cells by hybridization between antagomir-122 and endogenous miR-122 obtained a unequivocal success (Yu et al., 2015).

#### External Stimuli

Compared with the above microenvironment-responsive systems, external stimuli-responsive systems can be easily manipulated to precisely achieve spatiotemporal control and on-demand agents release (Lin et al., 2018). The main drawback of intrinsic stimuli is that internal environment of body is complicated and unmanageable, especially for the highly heterogeneous tumor tissues, which maybe results in an uncontrolled release (Thorat et al., 2019a).

Among the various light stimuli, near-infrared (NIR) has the great advantages of minimal absorption and deep penetration into tissue (Wu et al., 2015; Sun et al., 2017). The NIR-I (700–950 nm) and NIR-II (1000–1350 nm) are the most widespread regions for light-responsive systems. The MSNs hybridized with photoabsorbing materials exploit the fine photothermal effect limited within tumor tissues through converting light energy to thermal energy, which is a particularly promising phenomenon for use in light-responsive agents release (Figure 4C; Lee et al., 2016). NIR-thermal agents precision delivery can be reasonably attributed to the following reasons: first, the Brownian motion of agents would be accelerated by the photothermal effect; second, the photothermal effect could increase tumor cells membrane permeability; third, the photothermal effect may destabilize the membrane of endosome and thus facilitate escape of the agent from the endosome (Lee et al., 2016; Shao T. et al., 2016; Chen et al., 2018).

Besides aforementioned applications, M-MSNs are not only for magnetic-mediated targeting, but also for stimtli-responsive release. Alternating magnetic field (AMF) can heat M-MSNs by the magnetothermal effect, and then, the thermal fluctuations within M-MSNs trigger agents release (He and Shi, 2014; Wang et al., 2018). The mechanisms of the magnetothermal effect by these nanoparticles are related to brownian motion, neel relaxation, and hysterysisloss. And the release profile of agents can be regulated by changing the field strength and frequency of AMF. By the way, magnetic fluid hyperthermia will cause damage on tissues surrounding the nanoparticles to kill cancer cells. But for safety of healthy tissues, combination of field amplitude of about 10 kA m^–1^ and a frequency of about 400 kHz was suggested for stimulation (Thorat et al., 2019a).

In the future, with advance of nanotechnology, more various stimuli-responsive will be realized in MSNs for precision delivery.

## MSNs in HCC Detection and Diagnosis

### MSNs-Assisted Bioimaging

Ultrasound (US) is still the most common technique to screen the HCC since it is safe, economical and accessible (Verslype et al., 2012). However one study reported that the sensitivity of US for the small lesion (<2 cm), which is important for early detection and diagnosis of HCC, is only 27.3% (Kim et al., 2017). To improve diagnostic accuracy and sensitivity for early stage tumor, photoacoustic (PA) imaging integrating optical and ultrasound advantages have been developed which has the highest resolution in deep tissue compared with any conventional imaging tools (Liu Y. et al., 2019). So in the integrated system, PA imaging easily combined with ultrasound imaging can strongly enhance the clinical diagnosis (Kim et al., 2016). To this end, Lee et al. synthesized a MSNs-based liver targeting PA contrast agent, hyaluronate–silica nanoparticle (HA–SiNP) conjugate. Because of strong photoacoustic signal of SiNP in NIR windows, the PA amplitude in liver after HA–SiNP conjugates injection was remarkably enhanced 95.9% compared to normal liver beyond other PA contrast agents, which provides more detail anatomical and functional information for HCC diagnosis (Lee et al., 2018).

Before treatment, dynamic contrast-enhanced magnetic resonance imaging (MRI) is considered as the best approach to define the tumor staging and assist in choosing a suitable treatment strategy. Non-specific contrast agents, Gd complexes, have been the most widely applied agents for liver MRI (Bellin et al., 2003). Nevertheless, for HCC diagnosis, liver-specific MRI contrast agents, which mainly target the liver tissue, maybe an alternative choice. Aim to make up the limitations of non-specific contrast agent, Kim et al. investigated the liver-specific MRI contrast agent, Mn^2+^-doped SiO_2_ nanoparticles (Mn-SiO_2_), enhancing the visibility of HCC lesion. The nanoparticles engulfed in Kupffer cells would release the Mn^2+^ ions, thus T1-weighted MRI shows hyperintense in healthy liver tissues with abundant Kupffer cells over lesions, which are always lack of Kupffer cells (Kim et al., 2013).

Furthermore, it has been reported that intraoperative fluorescent imaging for imaging-guided surgery by virtue of MSNs could dramatically improve surgical intervention of tumor (Zeng et al., 2016). This RGD-conjugated MSN highly loaded with ICG dye could precisely delineate the margins of HCC intraoperatively by NIR. Depend on the subjective experience, only the conspicuous tumors (5.09 ± 2.31 mm) could be visually discriminated by surgeons intraoperatively. This lesion is also confirmed by fluorescent imaging. The microtumor lesions (0.4 ± 0.21 mm), which could not be recognized with the nake eye, are accurately detected by fluorescent imaging. Currently in operations, surgeons mainly rely on conventional preoperative imaging methods and subjective experience. However, in most cases, tumor microfoci can’t be discovered which is regarded as one of the etiology for tumor recurrence. But in the intraoperative fluorescent imaging by MSNs, tumor microfoci in liver could easily be distinguished and resected resulting in better surgical outcomes. It is helpful to reduce the high postoperative recurrence rate of HCC.

### MSNs-Assisted Liquid Biopsy

Liquid biopsy refers to non-invasive tests analyzing the bodily fluids and is a promising option to detect early stage HCC (Zhou et al., 2016; Ye et al., 2019). The source from tumor for liquid biopsy covers circulating tumor cells (CTCs), nucleic acids, proteins and circulating exosomes (Lee et al., 2018). Currently, several new MSNs have been reported for detection of tumor cells and their associated molecules. Hu et al. developed functionalized MSNs for specifically detecting HCC cells with assist of a biotin-labeled aptamer. The binding rate with hepG2 cells could reach approximately 90%, while the binding rate with L02 cells was close to 2%, which means the nanoparticles established a sensitive detection system for HCC cells (Hu et al., 2017). In another research, MSNs are utilized to detect the apoptotic tumor cells for evaluation of treatment response. This detection system contains two steps: (1) the HCC cells among various cells would be immobilized on the nanotubes; (2) the apoptotic HCC cells would be quantitated through the specific interaction between antiphosphatidyl serine antibody and phosphatidylserine. This cytosensor has a high sensitivity, which even could respond as low as 800 cells mL^–1^ (Wu et al., 2012). Besides, MSNs are also used to enrich phosphopeptides from serum of HCC patients. And then the phosphopeptides could be extract from MSNs for further analysis (Hu et al., 2009).

## MSNs in HCC Treatment

### Drug Therapy

Systemic chemotherapy usually is the only option for patients in advanced cancer, however, no satisfactory results have been obtained in HCC (Bruix et al., 2015; Gao J. et al., 2015). Thus, numerous studies focusing on MSNs to improve the drug effect have been reported. Awing to the unique structure of MSNs, they are suitable for delivery of both hydrophobic/hydrophilic anticancer drugs. Moreover, drugs release in MSNs always experience a decrease in release rate, resulting in sustained release pharmacokinetics (Kumar et al., 2015; He et al., 2017). DOX, which is easily tracked through fluorescence effects, is widely used as a model drug for assessing drug loading and delivery capacity in MSNs (Rudzka et al., 2013; Xie et al., 2014; Yang et al., 2015). Although DOX in MSNs had showed a good antitumor effects, other drugs that is more sensitive for HCC should be explored. It’s worth noting that many hydrophobic drugs loaded in MSNs overcome their poor water solubility, including paclitaxel (Li et al., 2010; He et al., 2017; Xu et al., 2017), curcumin (Lv et al., 2017; Xing et al., 2018), berberine (Yue et al., 2018b), and so on. Co-delivery multiple drugs have been recognized as a more efficient treatment than a single drug. Thus a MSNs-based nanoparticle had been developed for co-delivery of SO and ursolic acid (UA). Compared with the SO or UA respectively treated group, the expression of EGFR and VEGFR2 in SO + UA treated group decreased about 60%, which tremendously increase apoptosis of tumor cells and inhibit proliferation, adhesion, migration and angiogenesis. The further *in vivo* increased therapeutic efficacy of nanoparticles demonstrate the synergistic effect of SO and UA (Zhao et al., 2017). UA, which possesses significant antitumor activity, is limited in clinical application with its poor water solubility. By virtue of MSNs, UA can be delivered to tumor tissues, exhibiting a synergetic antitumor effect with SO. It suggested a promising approach for exploiting the potential of the drugs.

### Protein Therapy

Proteins have been explored as potential therapeutic candidates in cancer therapy since they have a low amount of side effects and the immunity to multidrug resistance mechanism (Epler et al., 2012; Deodhar et al., 2017). However, due to their facile degradation and fragile structure *in vivo*, effective delivery of proteins is a great challenge in spite of the vehicles (Escoto, 2013; Deodhar et al., 2017). Thus, MSNs have been used as promising vehicles to deliver proteins in HCC therapy (Zhang et al., 2014; Liu et al., 2017). The porous and stable nature of MSNs allows proteins encapsulated inside nanoparticles and provides a stable shelter to protect proteins. Lipid bilayer-modified MSNs had been designed to deliver ricin toxin A-chain (RTA) in which a few of RTA prematurely release and the activity of the remanent proteins was retained. RTA-loaded MSNs induce apoptosis in Hep3B at picomolar concentrations of RTA, which is 3500-fold less than the IC50 values of free RTA. These excellent functions enable protein-based therapies to reach their full potential (Epler et al., 2012). Covalent modification of protein on the MSNs surface was another approach for protein delivery. The proteins in this MSNs release via cleavage of the covalent bond in the pH/redox stimulation. Moreover, the proteins immobilized on the surface can be used as gatekeepers mentioned previously since the hydrodynamic diameters of the proteins are sufficient to cover the pores of MSNs. Relatively, in this way, the protective effects for proteins may be weaker (Pei et al., 2018).

### Gene Therapy

Gene therapy has been regarded as a new opportunity to satisfy the needs in treatment of cancer (Das et al., 2015). With the advancement in RNA biology, gene therapies not only introduce the exogenous genes by DNA but also change the gene expression at the mRNA level through by virtue of short interfering RNAs (siRNAs), miRNAs and antisense oligonucleotides (ASOs) (Shim et al., 2018). Recently, Clustered regularly interspaced short palindromic repeats/CRISPR-associated nuclease 9 (CRISPR-Cas9) also opens a new avenue in gene therapy to correct the mutations of cancer (Karimian et al., 2019). However, the development of an efficient and safe vector for therapeutic genetic materials is still a major issue. MSNs are promising carriers for gene delivery for their versatile payload of various genetic materials without chemical modification. For forming a stable complex with electronegative nuclei acid, MSNs are often modified to possess net positive charges by methods including amination-modification (Xiao et al., 2010; Yu et al., 2015; Zheng et al., 2018) and cationic polymer functionalization (Xue et al., 2017; Wang et al., 2018). In these terms, the modified surface can not only increase the adsorption capacity of negatively charged nuclei acid molecules, but can also facilitate MSNs to escape from endosome/lyposomes by “proton sponge effect” (Tang et al., 2012; Wang et al., 2018).

To overcome multidrug resistance in HCC, Xue et al. prepared lipid-coated MSNs containing DOX and miR-375 which can inhibit P-glycoprotein (P-gp) expression via inhibition of astrocyte elevated gene-1 (AEG-1) expression in HCC. P-gp, which is overexpressed in multidrug resistance cells in HCC, could impede the effectiveness of chemotherapy. So augment the level of miR-375 in HCC cells would serve as a credible way to overcome multidrug resistance. Further evaluation of antitumor effect in the DOX-resistant HepG2 cells xenograft tumor mouse model showed the tumor volume in DOX and miR-375 nanoparticles treated group is only about half of that in DOX nanoparticles treated group in 1 month, suggesting an alternative option to overcome multidrug resistance in HCC by these nanoparticles (Xue et al., 2017).

### Phototherapy/Sonodynamic Therapy (SDT)

Recently, phototherapy has emerged as a promising strategy for HCC. Photothermaltherapy (PTT) and photodynamic therapy (PDT) are two main types of phototherapy. PTT destroies the tumor cells by light-induced photothermal effects, while PDT damages the tumor cells by light-induced cytotoxic singlet oxygen (^1^O_2_), one kind of the most representative ROS (Liu H. et al., 2011; Zhao et al., 2012). NIR is widely used in PTT and PDT because of its superiority compared to other lights. In recent reports, MSNs prepared with photothermal agents including MoS_2_, C, Au, CuS and indocyanine green (ICG) showed a strong photothermal effect (Wu et al., 2015; Lee et al., 2016; Wang Z. et al., 2016; Wang et al., 2017a; Chen et al., 2018). Usually normal cells possess a higher heat tolerance over cancer cells at elevated temperatures around 43°C, so the heat generated (43^°^C) would trigger the death of tumor cells only (El-Boubbou, 2018). Gao B. et al. (2015), reported MSNs with gold core acted as a radiation sensitizer, thereby inducing the more effective radiotherapy by iodine 125 seed. Furthermore, Wang et al. (2019) rethink that radiosensitization strategy is not sufficient because of the hypoxic microenvironment in HCC, so they fabricated Janus-structured gold triangle-mesoporous silica nanoparticles to ameliorates hypoxia through PTT generated by gold triangle. This research indicated synergistic radio-photothermal therapy is a reasonable combination scheme. And there are some photosensitizers, such as Zinc(II)-phthalocyanine, Chlorin e6, Photosan-II loaded in MSNs for PDT in HCC (Liu et al., 2014; Lv et al., 2015; Lin et al., 2018). The success of PDT depends on the potential of photosensitizers to transfer energy from light to tumor dissolved oxygen (O_2_) to generate ^1^O_2_. Pre-existing hypoxia in HCC and O_2_ consumption during PDT can remarkably lower down the PDT efficacy (Thorat et al., 2019a). To address this problem, *in situ* synthesized Pt nanoparticles as a catalyst to convert H_2_O_2_ into O_2_ on MSNs was constructed. And 4.2-fold O_2_ and 1.6-fold ^1^O_2_ generation ability compared to normal MSNs greatly improved the PDT efficacy in HCC and exhibited threefold tumor suppression ability in HCC mice model (Lan et al., 2019).

However, when tumor locates in the deeper tissue, which is common for HCC, the lights arriving to the tumor are so weak that hardly produce an enough cytotoxic effects. Therefore, MSNs-based SDT has been developed account for the advantage of deeper tissue-penetrating of ultrasound (Li et al., 2018b). In spite of the ROS cytotoxic effects on the tumor cells, moreover, SDT can enhance the chemotherapeutic sensitivity of tumor cells by activating the cellular internalization and the mitochondrial apoptotic pathway, and inhibiting ATP-binding cassette transporter (Xu et al., 2013; Qian et al., 2016). For example, Li et al. (2018b) chose MONs as nanocarriers for the delivery of both sonosensitizer (protoporphyrin, PpIX) and DOX. The tumor-inhibiting rates in mice increased about 21.6% with the US irradiation (Li et al., 2018b). So the combination of SDT and chemotherapy is a hopeful strategy for HCC treatment.

## Dual Effects of Therapy and Diagnosis in MSNs

**FIGURE 5:**
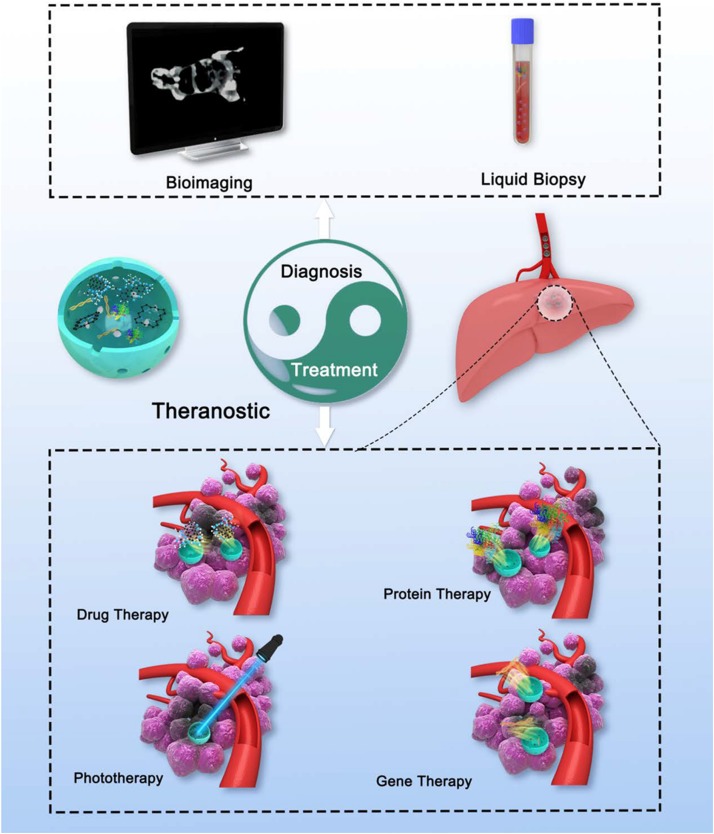
Schematic illustration of theranostic MSNs. MSNs with rational design could integrate various diagnosis and treatment function, which is similar to Tai Chi all-embracing.

As described, MSNs-based therapy and diagnosis for HCC have been investigated a lot separately. More importantly, MSNs-based theranostic nanostructures are capable of detecting the tumor not only before or after, but also during the treatment anytime. Recently, Chen H. et al. (2017) offered a proposal extending the concept of nanotheranostics from nanomedicine owning both diagnostic and therapeutic functions, to the approaches that use diagnosis to aid nanoparticle therapy procedures. With such approaches, medical treatments can be tuned promptly on the basis of the detection results, which means more specific therapies for individual patient (Figure 5; Xie et al., 2010).

The easiest way to achieve theranostics is co-delivery of therapeutic and imaging agents. Ashley et al. modified MSN with supported lipid bilayers resulting in nanostructures (“protocells”), which could be loaded with mixtures of therapeutic (drugs, proteins, genes) and diagnostic (quantum dots) agents (Ashley et al., 2011). Quantum dots as a fluorescent dyes could trace the biodistribution of content in nanostructures because mixtures of therapeutic agents and quantum dots will be released from nanostructures simultaneously. Within a certain period of time, quantum dots remaining in nanostructures could show the location of nanoparticles targeting to tumor for diagnosis. Therefore, in this way, the fluorescent dyes mainly tracked the therapeutic agents in tumor rather than diagnosis for HCC. The meaning of this monitoring is to investigate tumor accumulation and release of agents, which is an important factor for nanoparticle therapy procedures. On the contrary, if diagnostic agents stay in nanoparticles throughout, the diagnosis and therapy for tumor could be implemented at the same time (Fan et al., 2019).

In another way, because many nanomaterials are already imaging agents, hybrid nanostructures fabricated with these nanomaterials and MSNs could make diagnosis and therapy together. Among all hybrid nanostructures, the physically responsive (light/magnetic/ultrasonic) nanostructures widely explored as innovative “theranostics” in cancer have been described in excellent reviews (Thorat et al., 2019a, b). The greatest strength for these nanostructures is that their properties to physically stimulus accord with the diagnosis mode (MRI/US) in clinic. Li et al. successfully coat a mesoporous-silica layer onto the surface of Ti_3_C_2_ (Ti_3_C_2_@mMSNs). Ti_3_C_2_ has a high photothermal-conversion efficiency and enables Ti_3_C_2_@mMSNs to possess the potential contrast-enhanced PA-imaging and heat production property. So these nanostructures can monitor the photothermal hyperthermia treatment process in real-time (Li et al., 2018c). In another study, Liu et al. prepared the capping MSNs-coated iron oxide nanoparticles with programmable DNA hairpin gates to form M-MSNs, which could decrease T_2_-weighted tumor signal in MRI for HCC diagnosis. Interestingly, BHQ1 (fluorescence quencher) and 6-carboxyfluorescein (FAM) were linked to the tail extension of the DNA hairpin structure. When DNA hairpin gates change the conformation after addition of HCC- specific miRNA-21, the fluorescence of FAM will significantly “ON” to monitor the release of DOX in nanostructures (Liu J. et al., 2019). In spite of the therapeutic functions, the above nanostructures have two diagnostic functions: positioning tumor tissues and monitoring drug release.

The most amazing theranostic nanostructure is developed using mesoporous silica layer as shells and up-conversion luminescent (UCL) GdOF:Ln (Ln = 10%Yb/1%Er/4%Mn) as cores by Lin et al. Under NIR irradiation, GdOF:Ln could efficiently transfer NIR energy to the conjugated PDT agent (ZnPc) and emit bright red up-conversion emission. The shell decorated with carbon dots also can generate photothermal effect at the same time. Gd/Yb has the strong X-ray attenuation endowing nanostructures for computed tomography (CT) contrast agents, meanwhile Gd-based particles can be harnessed as contrast agents for MRI. This nanostructure with excellent and rational design is appropriate for both various imaging (UCL, CT, MRI, PT) and various therapies (PDT, PTT, chemotherapy), thus achieving multimodal imaging guided combination therapies (Lv et al., 2015).

With the development of nanotheranostics, many researchers believe that the integration of diagnosis and therapy to single modality would really benefit patient over independently managed diagnosis and therapy (Kelkar and Reineke, 2011). However, recent development of theranostics in MSNs was limited to “how can theranostics modalities in nanoparticles be realized” rather than “why can theranostics modalities in nanoparticles benefit the patients.” In hospital, patients have no opportunity to occupy the diagnostic tools all the time, so the concept of real-time monitoring translating to clinic may be only few hours monitoring time to examine patients totally. And multimodal imaging provides good alternatives for patients, but we should weigh advantages and disadvantages. It is because that the more imaging modes nanoparticles can be applied in, the more complicated structure nanoparticles possess. In most cases, four or more imaging modes in nanostructures make no benefits to the patient compared to one imaging mode. We suggest that development of theranostic nanostructures should be based on clinical demand and clinical practice, which is the key for translational medicine.

## Conclusion and Outlook

In this review, we summarized the recent progress in HCC theranostic applications based on MSNs. We generalized the precision delivery strategies applied for HCC in recent researches, including passive targeting, active targeting and stimuli-responsive release. The multiple therapy and diagnosis approaches had been realized in MSNs. Among a large amount of applications, we highlighted theranostic hybrid nanostructures, in which combination of the modalities of diagnostic imaging and therapy endowed MSNs-based nanostructures the ability to image and monitor the tumor tissue during treatment making possible more timely adjustment of therapy.

Despite the extensive researches, some emerging therapy and diagnosis approaches have not been used in MSNs for HCC. Most recently, cancer immune therapy is growing obviously, including cancer vaccinations, chimeric antigen receptor (CAR) T-cell therapy and immune checkpoint blockade therapy (Zhang and Chen, 2018). Actually, many MSNs-based immune therapies have been reported in other cancers (Ding et al., 2018; Li et al., 2018a; Xie et al., 2019). In addition, a lot of clinical trials about immune checkpoint blockade therapy such as PD-1 therapy for HCC are underway. Therefore, immune therapy is a potential development point for MSNs-based HCC therapy. Positron Emission Tomography (PET) is important for metastasis and prognostic assessment in patients with HCC (Filippi et al., 2019). Some Radiolabeling MSNs also have been reported for PET (Ni et al., 2018), thus it is hopeful to accomplish for more accurate diagnosis of HCC due to its smart recognition to tumor cells.

Although the preclinical trials of MSNs are successfully completed, currently, there are no MSNs that have been approved applied in clinic. There remain several critical challenges that need to be overcame for MSNs. First, the current small animal models are not suitable to evaluate the delivery efficiency and long-term toxicity of nanoparticles in humans. Second, the laboratory scale production of MSNs cannot easily repeat in the industrial scale of production for clinical application, especially for the complicated modified MSNs. Third, there is no thorough evaluation criterion, which may confuse the researchers to improve the existing MSNs. Nevertheless, there are some clinical trials in silica nanoparticles, the prospect of clinic translation for MSNs was less than good. We should focus our efforts in the following aspects: (1) Try to use big animals to assess safety and efficacy of the MSNs; (2) Improve and simplify production process of MSNs; (3) Establish a homogenized evaluation system; (4) modulate the theranostic functions of MSNs closer to the clinic. The long-wind road for clinical translation and commercialization of MSNs still needs researchers going on.

## Author Contributions

XX brought forward the subject and guided the writing. YT gathered the research data and wrote the manuscript. JW supervised the writing and revising. All authors critically revised the manuscript and approved it for publication.

## Conflict of Interest

The authors declare that the research was conducted in the absence of any commercial or financial relationships that could be construed as a potential conflict of interest.
